# BACHD Mice Recapitulate the Striatal Parvalbuminergic Interneuron Loss Found in Huntington’s Disease

**DOI:** 10.3389/fnana.2021.673177

**Published:** 2021-05-24

**Authors:** Vyshnavi Rallapalle, Annesha C. King, Michelle Gray

**Affiliations:** ^1^Department of Neurology, Center for Neurodegeneration and Experimental Therapeutics (CNET), University of Alabama at Birmingham, Birmingham, AL, United States; ^2^Department of Clinical and Diagnostic Sciences, Undergraduate Biomedical Sciences Program, University of Alabama at Birmingham, Birmingham, AL, United States; ^3^Graduate Biomedical Sciences, Neuroscience Theme, University of Alabama at Birmingham, Birmingham, AL, United States

**Keywords:** Huntington’s disease, striatum, interneuron, parvalbumin, somatostatin, calretinin, cholinergic

## Abstract

Huntington’s disease (HD) is a dominantly inherited, adult-onset neurodegenerative disease characterized by motor, psychiatric, and cognitive abnormalities. Neurodegeneration is prominently observed in the striatum where GABAergic medium spiny neurons (MSN) are the most affected neuronal population. Interestingly, recent reports of pathological changes in HD patient striatal tissue have identified a significant reduction in the number of parvalbumin-expressing interneurons which becomes more robust in tissues of higher disease grade. Analysis of other interneuron populations, including somatostatin, calretinin, and cholinergic, did not reveal significant neurodegeneration. Electrophysiological experiments in BACHD mice have identified significant changes in the properties of parvalbumin and somatostatin expressing interneurons in the striatum. Furthermore, their interactions with MSNs are altered as the mHTT expressing mouse models age with increased input onto MSNs from striatal somatostatin and parvalbumin-expressing neurons. In order to determine whether BACHD mice recapitulate the alterations in striatal interneuron number as observed in HD patients, we analyzed the number of striatal parvalbumin, somatostatin, calretinin, and choline acetyltransferase positive cells in symptomatic 12–14 month-old mice by immunofluorescent labeling. We observed a significant decrease in the number of parvalbumin-expressing interneurons as well as a decrease in the area and perimeter of these cells. No significant changes were observed for somatostatin, calretinin, or cholinergic interneuron numbers while a significant decrease was observed for the area of cholinergic interneurons. Thus, the BACHD mice recapitulate the degenerative phenotype observed in the parvalbumin interneurons in HD patient striata without affecting the number of other interneuron populations in the striatum.

## Introduction

Huntington’s disease (HD) is caused by a CAG repeat expansion in the gene that encodes for the huntingtin (HTT) protein, resulting in an expanded polyglutamine repeat (MacDonald et al., 1993). The HTT protein is expressed in all cells in the brain as well as other cells within the body. However, the striatum is the most affected region of the brain in HD disease progression, as it undergoes significant and progressive atrophy and neuron loss (Reiner et al., 2011). The GABAergic medium spiny neurons (MSN) undergo the most degeneration (Han et al., 2010). Cortical projection neurons and other areas of the brain are also affected, but to a lesser extent (Cudkowicz and Kowall, 1990). MSNs are the major input neuron in the striatum as well as the sole output neuron and are central to controlling motor function, and their degeneration contributes to abnormalities associated with HD (Crossman et al., 1988; Berardelli et al., 1999). The activity of these MSNs is regulated by multiple inputs including from extrastriatal glutamatergic and dopaminergic neurons, intrastriatal cholinergic interneurons, and intrastriatal GABAergic interneurons (Gerfen and Bolam, 2016).

There are numerous types of principal striatal interneurons that can make up this 5%, including the Fast-spiking interneuron (FSI) expressing parvalbumin (PV), low threshold spiking (LTS) expressing somatostatin/neuropeptide Y/nitric oxide synthase (SOM/NPY/NOS) interneuron, choline acetyltransferase (ChAT), calretinin (CR), and tyrosine hydroxylase (TH) expressing interneurons (Tepper et al., 2010; Klug et al., 2018). These different interneurons interact with MSNs through various somatodendritic locations to modulate their function (Ibanez-Sandoval et al., 2010, 2011; Xenias et al., 2015; Munoz-Manchado et al., 2016). There have been multiple electrophysiological studies in mutant HTT (mHTT) expressing mice demonstrating abnormalities in these various interneuron populations that contribute to increased inhibition of the MSNs. In the R6/2 mouse model, stimulation of PV+ cells resulted in significantly larger GABA-mediated responses in MSN than what was observed after stimulation of PV+ cells in wild-type mice. In the same study, PLTS/SOM+ interneurons fired more action potentials in mHTT-expressing R6/2 and BACHD transgenic mice (Cepeda et al., 2013). Another study in the Q175 knock-in mouse model found that PV+ interneurons showed greater excitability as the disease progressed, and a decrease in dendritic branching and synaptic inputs onto PV+ interneurons (Holley et al., 2019b). The SOM+ interneurons in the Q175 knock-in mice showed similar electrophysiological properties to those seen in the R6/2 mouse model (Holley et al., 2019a). Thus, there are some intriguing electrophysiological changes observed in both FSI/PV+ and PLTS/SOM+ interneurons in HD mouse models that could explain the increased inhibitory input on MSNs in the various HD mouse models.

There have been a few studies examining some of these interneuron types in striatal tissue from HD patient brains. In assessing the numbers of the various interneuron types in the striatum, one study only identified the PV interneuron as significantly diminished in abundance when compared to controls (Reiner et al., 2013). The study also revealed that the abundance of striatal PV+ cells continued to significantly decrease as HD progressed from early stages (Grade 1) to late stages (Grade 4; Reiner et al., 2013). The numbers of SOM+/NPY+ possessing interneurons were not significantly altered. Studies on calretinin expression and the abundance of calretinin-positive interneurons have found that these interneurons are largely unaffected in striatal tissue from HD patients (Reiner and Deng, 2018). Similarly, cholinergic interneurons were not decreased in number in HD patients’ striatal tissue (Reiner and Deng, 2018); however, studies in the Q140 mHTT expressing mouse model showed that despite a normal number of cholinergic interneurons, the somas of these cells were smaller, with fewer and thinner dendritic projections when compared to WT mice (Deng and Reiner, 2016). Another study using the Q175 mHTT expressing mouse model showed a decrease in cholinergic interneuron dendritic complexity (Deng et al., 2021). In this study we used 12–14 months (mos) old BACHD and wild-type mice. At 12–14 mos of age, the conditional human mHTT expressing BACHD mouse model displays motor and psychiatric-like abnormalities, neuropathological changes, and significant electrophysiological deficits in MSN as well as striatal interneurons (Gray et al., 2008; Wang et al., 2014; Wood et al., 2019; King et al., 2020). Due to the prominent nature of the striatal MSN electrophysiological deficits in the BACHD mice (Gray et al., 2008; Andre et al., 2011a,b; Cepeda et al., 2013; Plotkin et al., 2014; Wood et al., 2019), we sought to determine whether the BACHD mouse model at 12–14 mos of age recapitulates the changes seen in interneurons in HD patient tissue.

## Materials and Methods

### Animals

All of the experimental procedures performed on mice in this study were approved by the University of Alabama at Birmingham’s (UAB) Institutional Care and Use Committee and were in accordance with the National Institutes of Health Guide for the Care and Use of Laboratory Animals. All of the mice used in this analysis were 12–14 mos old, and both male and female mice were used.

### Perfusion and Sectioning of Mouse Brain

Mice were perfused intracardially with 4% paraformaldehyde in 0.01 M phosphate-buffered saline and the brains removed. The brains were incubated overnight at 4°C in 4% paraformaldehyde and then incubated in 30% sucrose for cryoprotection. Next, the brains were frozen on dry ice and cut into 40 μm coronal sections on a Leica SM2010R microtome, and the sections were placed in cryoprotectant (0.01 M PBS, formula) before storage at −20°C. The first section containing striatum was identified and placed in the first well of a 24-well plate, then the next nine serial sections were placed in the next nine wells. Next, the 11th section is placed in the first well and the next nine wells placed in the subsequent wells, such that the first 10 wells contain four sections each, where each section in the well is separated by 400 μm. A total of 14 wild-type and 14 BACHD mice were used for the experiments performed: five wild-type (3 M, 2 F) and 5 BACHD (2 M, 3 F) mice were analyzed for parvalbumin expression, three wild-type (2 M, 1 F) and three BACHD (2 M, 1 F) mice were analyzed for somatostatin expression, three wild-type (2 M, 1 F) and three BACHD (1 M, 2 F) were analyzed for calretinin expression, and three wild-type (1 M, 2 F) and three BACHD (1 M, 2 F) were analyzed for ChAT expression.

### Indirect Immunofluorescent Staining

**Figure 1:**
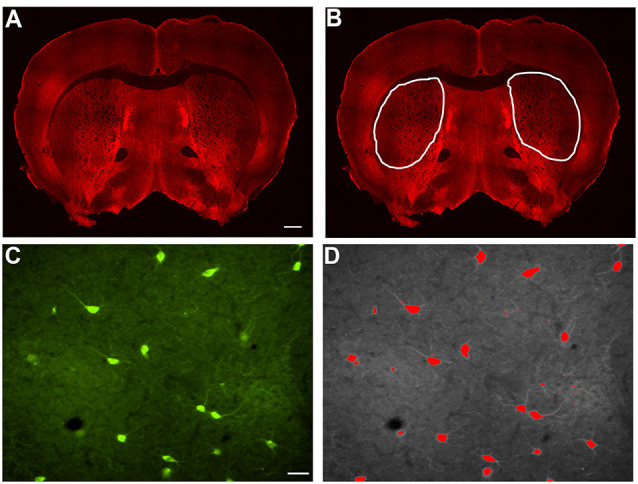
Example of indirect immunofluorescence and thresholding of image. **(A)** A stitched image of indirect immunofluorescent staining of SOM+ interneurons in a coronal section containing rostral striatal tissue. In order to count the positively stained interneurons, a region of interest was drawn around each striatal half **(B)**, and the number of cells per half were counted and analyzed as number/1 × 10^6^ μm^2^ striatal area in Figures 2C, 3C, 4C, and 5C. Indirect immunofluorescent staining of ChAT+ cells in a 20× image is shown **(C)**. The thresholding method was utilized to identify only the cell bodies above the threshold, while excluding the processes, in each 20× image **(D)**. Only cell bodies with processes within the same focal plane were analyzed in Image J Fiji. The average area of all of the cells in a single 20× image is represented as a single data point in Figures 2D, 3D, 4D, and 5D. Similarly, the average perimeter of all the cells in a single 20× image is represented as a single data point in Figures 2E, 3E, 4E, and 5E. The total number of cells analyzed for each interneuron type are represented in the results. Scale bar = 500 μm in **(A)** and scale bar = 100 μm in **(C)**.

Indirect immunofluorescent staining (Figure 1A) was used to visualize the four different interneurons. Coronal sections between Bregma levels +1.18 and 0.4 were washed with 0.01 M phosphate-buffered saline (PBS) three times for 10 min. Then, the sections were incubated for 1 h in a blocking solution which included 0.01 M PBS, 0.08% sodium azide, 3% bovine serum albumin, 2% normal goat serum, or normal donkey serum. Next, the sections were incubated in the blocking solution with 0.2% Triton-X 100 solution along with the corresponding primary antibody at different concentrations. The following primary antibody concentrations were used: parvalbumin (Millipore MAB572, 1/1,500), somatostatin (Millipore MAB354, 1/100), calretinin (Millipore MAB1568, 1/400), and ChAT (Millipore AB144, 1/100). The sections were then incubated overnight at 4°C on a shaker. The next day, the sections were washed with 0.01 M PBS in 3× 10 min increments. The sections were then incubated in the appropriate secondary antibody at varying concentrations for 2 h at room temperature on a shaker. The parvalbumin sections were incubated in an anti-mouse 488 secondary antibody (Invitrogen A11029, 1/500). The somatostatin sections were incubated in an anti-rat cy3 secondary antibody (Jackson ImmunoResearch 712-165-153, 1/500). The calretinin sections were incubated in an anti-mouse 488 secondary antibody (Invitrogen A11029, 1/400), and the ChAT sections were incubated in an anti-goat 488 secondary antibody (Invitrogen A11055, 1/500). The sections were rinsed again with 0.01 M PBS for 3 × 10 min, and they were mounted using VectaMount with DAPI.

### Cell Counting, Area, and Perimeter Analyses

Striatal interneurons were counted and measured for area and perimeter. In order to count the interneurons, bilateral images of the striatum from coronal sections were taken at 4× magnification on a Nikon Eclipse T*i*-S microscope. Cell counts were performed using ImageJ Fiji. Each of the individually labeled striatal interneurons was counted with the “Cell Counter” plugin. The total number of interneuron cell bodies labeled by each antibody bilaterally in each section was calculated in the region of interest that was defined in ImageJ Fiji by outlining the striatum (Figure 1B) and displayed per striatal side/1 × 10^6^ μm^2^ striatal area. To perform the area and perimeter analyses, images were taken at 20× magnification on a Nikon Eclipse T*i*-S microscope bilaterally through the entire striatum (Figure 1C). Three to five separate 20× images were chosen randomly and used to analyze the area and perimeter of the cells. The average area and perimeter of all of the cells in a given image were used as data points in the final analyses that are depicted in the figures. In ImageJ Fiji, the scale was set for each image based on image magnification to allow for calculating the area and perimeter of the cells. We applied the thresholding method to identify the signal in interneurons above the background, leaving only stained cell bodies, excluding processes that were above the threshold background in each image. Once the threshold was set, the “Analyze Particles” plugin was selected. The size and circularity options were adjusted and set to include each of the interneurons in the image and only neurons with a process in the same focal plane as the cell body were analyzed. “Overlay Masks” was used and the results for area and perimeter were obtained for each cell observed (Figure 1D). Using wild-type mice as a reference point, 75–80% of the interneuron types analyzed with this method, fell within the previously reported cell body sizes (data not shown). Based on no significant differences in counts between wild-type males and female mice, the data has been pooled.

### Scanning of Stitched Image

NIS Elements imaging software (version 4.51) was used to obtain the stitched image of a coronal section on the Nikon Eclipse T*i*-S microscope. The stitched image was acquired using a Nikon Plan Fluor 10×/0.30 (∞/0/17 WD 16) objective.

### Confocal Imaging

**Figure 2:**
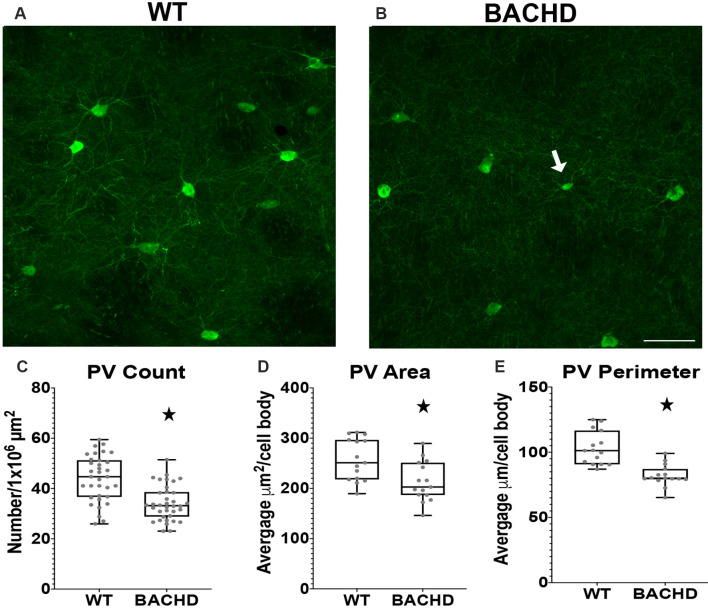
Parvalbumin interneuron numbers and sizes are decreased in the striatum of BACHD mice. Indirect immunofluorescence using an antibody to parvalbumin in the striatum of 12–14 months old WT **(A)** and BACHD **(B)** mice. The number (*p < 0.0001; **C**), area (*p = 0.0075; **D**), and perimeter (*p < 0.0001; **E**) of the PV+ neurons are shown. The Mann–Whitney test was used for the analyses. *n* = 5 WT and *n* = 5 BACHD. Scale bar is 50 μm.White arrow indicates a PV+ cell with decreased cell body area and perimeter.

**Figure 3:**
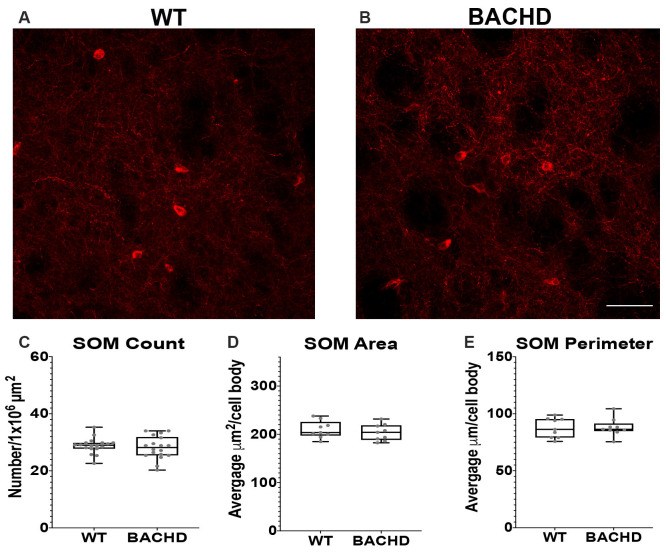
Somatostatin interneurons are not altered in BACHD mice. Indirect immunofluorescence using an antibody to somatostatin in the striatum of 12–14 mos old WT **(A)** and BACHD **(B)** mice. SOM+ neuron number (**C**; *p* = 0.4957), area (**D**; *p* = 0.5457), and perimeter (**E**; *p* = 0.9314) are shown. The Mann–Whitney test was used for the analyses. *n* = 3 WT and *n* = 3 BACHD. Scale bar is 50 μm.

**Figure 4:**
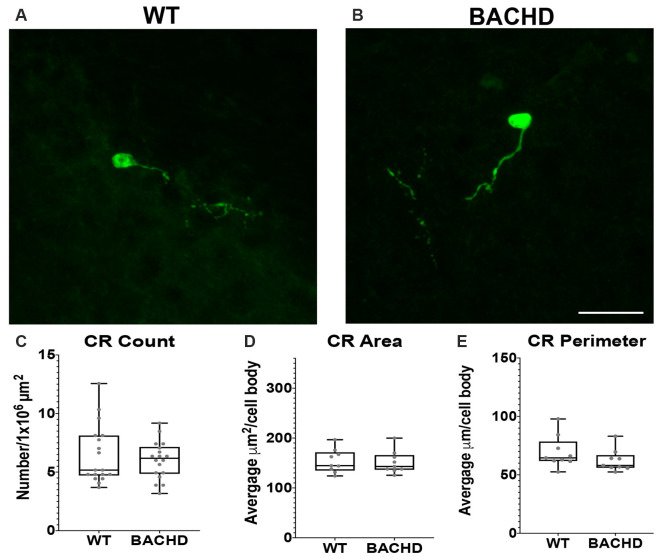
Calretinin (CR+) interneurons are not significantly reduced in BACHD mice at 12–14 months of age. Indirect immunofluorescent staining was performed on WT **(A)** and BACHD **(B)** mice to detect CR+ cells. CR+ cell number (*p* = 0.9314; **C**), area (*p* = 0.7304; **D**), and perimeter (*p* = 0.1903; **E**) are presented. The Mann–Whitney test was used for the analyses, and *n* = 3 WT and *n* = 3 BACHD. Scale bar is 25 μm.

**Figure 5:**
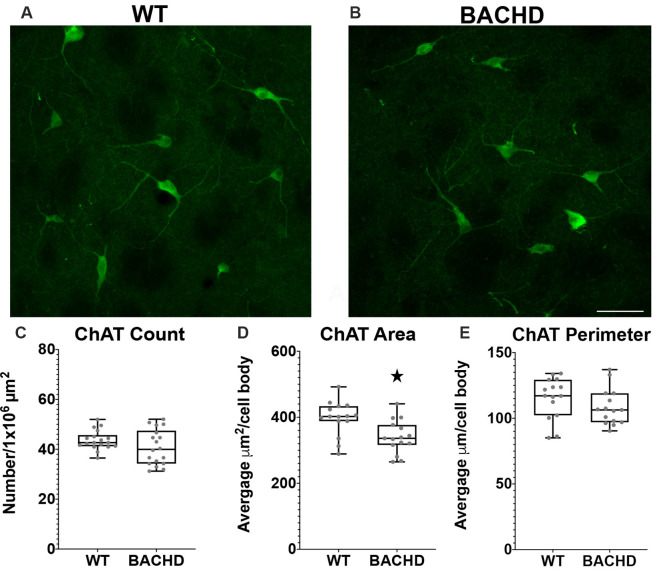
Cholinergic interneurons have decreased cell body area in BACHD mice at 12–14 months of age. Indirect immunofluorescent staining was performed on WT **(A)** and BACHD** (B)** mice with a ChAT+ antibody to detect cholinergic cells. ChAT+ cells were not significantly altered in number (*p* = 0.2482; **C**). The area **(D)** of BACHD ChAT+ cells was significantly decreased (*p = 0.0057) without significant reduction in the perimeter (*p* = 0.167) of the cells **(E)**. The Mann–Whitney test was used for the analyses, and *n* = 3 WT and *n* = 3 BACHD mice. Scale bar is 50 μm.

The Nikon Eclipse Ti2-C2 confocal microscope was used to acquire images displayed in Figures 2–5, and parameters in accordance with the Shannon–Nyquist theorem were followed for all images. The images were acquired using a Nikon Plan Fluor 40×/1.30 (OFM25 DIC H N2) oil immersion objective. The images shown are all from Z-stacks taken through 25–30 μm of tissue at z-step 0.5 μm.

### Statistical Analyses

The Mann–Whitney test was used to determine if there were any statistically significant differences in the cell numbers, area, and perimeter of the respective interneurons between wild-type and BACHD mice. A Box plot was used to display the distribution of the data among the genotypes based on the five number summary: minimum, lower quartile, median, upper quartile, and maximum. Each data point (one unit area; two per section) in the box plots for cell numbers represents the number of cells/unit area. For the area and perimeter of the cells, each data point represents the average for the cells contained in one 20× image. Statistical significance was set at *p* < 0.05. Analyses were performed using GraphPad/Prism.

## Results

### Parvalbuminergic Interneuron Number, Area, and Perimeter Are Decreased in the Striatum of BACHD Mice

Parvalbumin (PV+) containing GABAergic interneurons are medium-sized (10–25 μm) FSI and control the outputs of MSNs through a feedforward inhibition pathway (Hu et al., 2014). Studies using HD patient tissue showed that the decrease in PV+ cell number was progressive with the most robust changes found in HD patient tissue of higher grade (as defined by the percentage of striatal degeneration; Vonsattel and Difiglia, 1998) and greater disease progression. In addition, previous studies have demonstrated robust striatal atrophy and abnormal electrophysiological activity of PV+ interneurons (Giampa et al., 2009; Cepeda et al., 2013), therefore, we analyzed BACHD mice at 12–14 mos of age. We performed immunofluorescent staining using an antibody to analyze the number of parvalbumin positive (PV+) interneurons in BACHD mice (Figures 2A,B). We observed a significantly lower number of interneurons in BACHD mice (34.2 ± 7.0) when compared to WT mice (44.0 ± 9.1) per 1 × 10^6^ μm^2^ of striatal area (Figure 2C, *p* < 0.0001). The cell body area and perimeter of the remaining PV+ interneurons were also significantly decreased in BACHD mice (*p* = 0.0075 and *p* < 0.0001, respectively). BACHD mice (*n* = 244 cells) had an average area of 213.38 ± 99.37 μm^2^ and an average perimeter of 82.10 μm ± 39.47 while WT mice (*n* = 275 cells) had an average area of 257.28 ± 120.24 μm^2^ and an average perimeter of 103.15 ± 57.00 μm (Figures 2D,E). Therefore, BACHD mice PV+ interneurons exhibit the robust change in number that was observed in HD patient tissue.

### Somatostatin Interneuron Number, Area, and Perimeter Are Not Changed in BACHD Striatum

Another class of GABAergic interneurons is the medium–sized somatostatin (SOM)/neuropeptide Y (NY)/ nitric oxide synthase (NOS) containing interneurons (12–35 μm; Kawaguchi et al., 1995; Tepper and Koós, 2016). This class of interneurons is able to generate Ca^2+^ dependent low threshold spikes (LTS), and through a combination of excitatory and inhibitory signals, regulates the activity of MSNs (Figueredo-Cardenas et al., 1996). Numerous studies using various mouse models have shown that SOM+ interneurons have increased activity that could contribute to the increased GABAergic input onto MSNs that is seen in these mHTT expressing models (Gittis and Kreitzer, 2012; Cepeda et al., 2013; Holley et al., 2019b). Somatostatin interneuron numbers have not been shown to be altered in post-mortem HD patient tissue. Nonetheless, given the altered electrophysiology of SOM+ interneurons in BACHD mice, we also analyzed their numbers and cell body size at 12–14 mos of age (Figures 3A,B). We found that the number of SOM interneurons was not significantly reduced in BACHD mice (BACHD- 28.1 ± 4.0) when compared to wild-type mice (WT- 28.8 ± 2.7, *p* = 0.4957; Figure 3C). The area (WT- 209.93 ± 68.64 μm^2^ and BACHD- 203.80 ± 91.14 μm^2^, *p* = 0.5457; Figure 3D) and perimeter (WT- 87.49 ± 23.36 μm and BACHD- 88.06 ± 30.90 μm, *p* = 0.9314; Figure 3E) of the cell bodies in the BACHD mice (*n* = 136 cells) were not significantly reduced when compared to WT mice (*n* = 143 cells).

### Calretinin Interneuron Number, Area, and Perimeter Are Not Changed in BACHD Striatum

Calretinin (CR+) is a calcium-binding protein that defines another class of GABAergic interneuron (Parent et al., 1995; Tepper et al., 2018). In rodents, CR+ cells have medium–sized cell bodies (7–20 μm) and make up about 0.5% of all striatal interneurons (Kawaguchi et al., 1995; Petryszyn et al., 2014, 2018). Medium and large–sized CR+ interneurons are undiminished in size in human HD patient tissue (Massouh et al., 2008; Camillo et al., 2018). In order to determine if CR+ cells are affected in BACHD mice, we characterized these cells using a calretinin antibody to detect CR+ cells in WT and BACHD mice at 12–14 mos of age (Figures 4A,B). The CR+ interneurons were not significantly reduced in number (WT- 6.46 ± 2.5 and BACHD- 6.02 ± 1.6, *p* = 0.9314; Figure 4C), area (WT- 153.83 ± 59.22 μm^2^ and BACHD- 151.38 ± 46.76 μm^2^, *p* = 0.7304; Figure 4D), or perimeter (WT- 69.41 ± 22.71 μm and BACHD- 62.24 ± 18.58 μm, *p* = 0.1903; Figure 4E) in BACHD mice (*n* = 39 cells) when compared to WT (*n* = 50 cells). Interestingly, the striatal CR+ interneurons were mostly present on the medial and lateral edges of each respective striata. The CR+ interneurons were also the least abundant of the interneurons counted.

### Choline Acetyltransferase Positive Interneuron Cell Body Area Is Decreased in BACHD Mice

Cholinergic interneurons are characterized by their large somatic body size (20–50 μm). These interneurons are also defined by the expression of choline acetyltransferase (ChAT) and vesicular acetylcholine transporter (VaChT). The main function of these striatal cholinergic interneurons is to modulate the activity of MSNs *via* acetylcholine release (Pisani et al., 2007). Some studies have shown that there are abnormal cholinergic cell properties without any significant loss in cell number but with a significant reduction in cell area and fewer and shorter processes in mHTT expressing mouse models (Holley et al., 2015; Deng and Reiner, 2016; Deng et al., 2021). We used a ChAT antibody to visualize cholinergic interneurons in the mouse striata of WT and BACHD 12–14 mos old mice (Figures 4A,B). When compared to WT mice, the cholinergic interneurons were not significantly reduced in number in BACHD mice (WT- 43.7 ± 4.0 and BAC- 40.8 ± 7.1, *p* = 0.2482; Figure 5C). There was a significant reduction in cholinergic neuronal cell body area in BACHD mice (WT- 397.13 ± 151.20 μm^2^, *n* = 231 cells and BACHD- 340.52 ± 148.78 μm^2^, *n* = 201 cells, *p* = 0.0057; Figure 5D), while a decrease in cell perimeter in the BACHD mice was not statistically significant (WT- 115.50 ± 45.05 μm and BACHD-108.35 ± 43.02 μm, *p* = 0.167; Figure 5E).

## Discussion

MSN account for 95% of the striatal neuronal population and undergo the most prominent degeneration in HD (Vonsattel et al., 1985; Reiner and Deng, 2018). There are two types of MSNs in the striatum that make up the direct (dMSN) and indirect (iMSN) pathway projecting neurons. The dMSNs project to the internal segment of the globus pallidus (GPi) and the substantia nigra pars reticulata. The iMSNs project to the external segment of the globus pallidus (GPe; Eidelberg and Surmeier, 2011; Gittis and Kreitzer, 2012). The classic hypothesis of basal ganglia function posits that the direct pathway promotes movement, whereas the indirect pathway inhibits movement (Cui et al., 2013; Freeze et al., 2013; Vicente et al., 2016). Degeneration and dysfunction of these MSNs result in motor abnormalities. In HD, the balance between the glutamatergic input and GABAergic input onto MSNs is severely altered. Evidence from mHTT expressing animal models suggest that the changes in glutamatergic input on MSNs is biphasic, with increases in glutamate release in early stages of HD followed by decreases in later stages (Joshi et al., 2009; Andre et al., 2011a; Cepeda and Levine, 2020). Perhaps, in order to counteract the effects of increased glutamate release, GABAergic input onto MSNs is increased in early stages. Interestingly, as HD phenotypes progress in the animal models, glutamatergic input decreases while GABAergic input does not decrease, but rather remains elevated (Joshi et al., 2009; Cepeda and Levine, 2020).

While degeneration is most prominently observed for MSNs, there is increasing evidence of degenerative changes of PV+ interneurons in mHTT expressing mice and HD patients. This is interesting given that PV+ interneurons and MSNs have the capacity to form more mHTT containing aggregates and nuclear inclusions while the other interneurons develop very few (Kosinski et al., 1999; Meade et al., 2002; Deng et al., 2021). However, while somewhat debated, the general expression level of HTT in the striatal interneuron populations is low compared to the MSNs (Kosinski et al., 1997). Interestingly, in the BACHD mouse model, while there are degenerating neurons in the striatum as indicated by increased numbers of dark neurons, analysis of the total NeuN+ neuron number by unbiased stereological methods, did not reveal significant overt neurodegeneration (Gray et al., 2008). However, here we observe significant alterations in the number and cell body morphology of the PV+ interneurons. This decrease in PV+ neurons may not have been revealed in the analysis of total NeuN+ neurons due to the general abundance of NeuN+ cells. Electrophysiological studies of these PV+ cells in various mouse models reveal larger amplitudes of synaptic transmission from PV+ interneurons onto MSNs (Cepeda et al., 2013). A decrease in the number of these cells could help explain the larger amplitudes of synaptic transmission because the remaining cells may be trying to compensate for the overall decrease of PV+ interneurons. Furthermore, abnormalities in these cells could contribute to the motor dysfunction seen in HD since these cells provide a major source of GABA inhibition to the MSN, and without this inhibition, the MSNs will fire more frequently and lead to hyperkinetic movements (Hu et al., 2014). The dysfunction of the PV+ cells has been implicated in dystonia in human patients (Reiner et al., 2013). Even though chorea is the earliest and most prominent motor abnormality observed clinically in adult-onset HD patients, dystonia is observed later in disease progression (Gernert et al., 2000; Gittis et al., 2011; Reiner et al., 2013).

Although there are altered electrophysiological properties of SOM+ interneurons, including increased firing in some mouse models expressing mHTT, the number of these cells is not reduced in HD patient tissue (Reiner and Deng, 2018). In agreement with that data, our experiments in the BACHD mice did not find any diminishment of SOM+ interneuron numbers or alteration in the size of the cell body. However, we did not assess the morphology of the processes of these interneurons. Nevertheless, there need to be additional studies to determine whether the presence of mHTT within SOM+ interneurons are causing alterations in the activity of these neurons or whether this is due to interactions with other cell types which could ultimately contribute to abnormal activity of MSNs in HD.

Calretinin-positive interneurons constitute a small number of the interneurons in the mouse striatum (Petryszyn et al., 2014; Petrella et al., 2018). These CR+ interneurons are not diminished in number or size in the BACHD mouse model. This recapitulates the findings of other studies that have found no loss of these cells in HD (Reiner and Deng, 2018). In human striatal tissue, many large CR+ interneurons express both ChAT and calretinin; however, this is not seen in mice (Petryszyn et al., 2014). Thus, examination of these cell types based purely on marker expression in humans is complicated by this dual expression. Furthermore, their contribution to the overall circuitry could be why these cells remain largely intact in human HD striatal tissue since cholinergic interneurons survive in HD (Massouh et al., 2008; Reiner and Deng, 2018). Interestingly, striatal calretinin expression is increased in 3–6-month-old YAC128 mice (Czeredys et al., 2013). Therefore, it could be worthwhile to investigate if these findings are observed in other mouse models because if true, it would identify another population of striatal interneurons that could contribute to the increased GABA input onto the MSNs seen in mHTT expressing mouse models.

Cholinergic interneurons have been found to exert a greater effect on iMSNs than dMSNs; thus, their dysfunction could prove to be a key contributor to HD pathogenesis since iMSNs are the more affected of the two cell types early in HD (Reiner and Deng, 2018). Previous studies have found that the number of cholinergic striatal cells are undiminished in HD mouse models and human tissue, although there have been findings of reduced cell body size and dendritic branching (Holley et al., 2015; Deng and Reiner, 2016; Deng et al., 2021). Our findings support the results of these studies as we found no loss in cell number but a decrease in the area of the cell. Interestingly, it has also been reported that the levels of ChAT and VaChT are decreased in the R6/1 striatum (Smith et al., 2006). In R6/2 mice, there are greater inhibitory effects on striatal cholinergic interneurons (Holley et al., 2015). Both of these findings could lead to decreased release of acetylcholine from these cholinergic cells. Not only does this affect the MSNs’ function directly, but this decrease in acetylcholine could also affect the MSNs’ indirectly. Cholinergic interneurons possess VGLUT3 which exert a glutamatergic effect on PV+ cells, and this excitatory influence on PV+ cells could also be reduced if cholinergic interneurons are not functioning at optimal levels (Higley et al., 2011; Nelson et al., 2014). Therefore, the decrease in acetylcholine release could cause greater dysfunction in PV+ cells which could contribute to dysfunctional MSNs.

We found that PV+ cells are decreased in size and number in BACHD mice. The abnormalities in the PV+ cells are of particular interest because the number of MSNs in BACHD mice is not significantly altered. Therefore, the loss of PV+ cells could be a large contributor to the motor abnormalities that are seen in BACHD mice due to the loss of their input onto MSNs. Further studies of the striatal interneuron sub-types and their interactions with the MSNs in the striatal microcircuit will aid in our understanding of how these cells are ultimately contributing to the behavioral and physiological changes observed in HD.

## Data Availability Statement

The original contributions presented in the study are included in the article, further inquiries can be directed to the corresponding author.

## Ethics Statement

The animal study was reviewed and approved by Institutional Animal Care and Use Committee of the University of Alabama at Birmingham. The approval number is 20162.

## Author Contributions

MG designed the study. AK generated and perfused the mice, performed brain sectioning, performed statistical analyses, and edited the manuscript. VR performed the indirect immunofluorescent staining, cell counting, morphological analyses, and performed statistical analyses. VR and MG wrote the manuscript. All authors contributed to the article and approved the submitted version.

## Conflict of Interest

The authors declare that the research was conducted in the absence of any commercial or financial relationships that could be construed as a potential conflict of interest.
